# Bariatric Surgery as a Potential Risk Factor for Patulous Eustachian Tube: A Comprehensive Literature Review

**DOI:** 10.7759/cureus.115965

**Published:** 2026-09-08

**Authors:** Karamallah Almasri, Sayed H Almosawi, Amreen Mustafa, Noor Ali, Rabab Kamal

**Affiliations:** 1 Research, Sandwell and West Birmingham Hospitals NHS Trust, Birmingham, GBR; 2 Training and Academics, Salmaniya Medical Complex, Manama, BHR; 3 Emergency, Sandwell and West Birmingham NHS Trust, Birmingham, GBR; 4 Medicine, Royal College of Surgeons in Ireland - Bahrain, Manama, BHR

**Keywords:** bariatric surgery, eustachian tube dysfunction, glp-1 agonists, patulous eustachian tube, rapid weight loss

## Abstract

Patulous Eustachian tube (PET) is a condition characterized by abnormally persistent patency of the Eustachian tube, resulting in symptoms such as autophony, aural fullness, and otalgia, with weight loss increasingly reported as a potential risk factor. This review aimed to evaluate the association between bariatric surgery and PET. PubMed, Google Scholar, and Scopus were searched from inception to August 2026 for studies addressing PET, bariatric surgery, weight loss, and Eustachian tube dysfunction. Available evidence, predominantly from small observational studies and case reports, demonstrates an association between PET and rapid weight loss following bariatric surgery. Additionally, similar reports following glucagon-like peptide-1 (GLP-1) agonists and severe malnutrition were observed. Bariatric surgery cannot currently be established as an independent cause of PET. Prospective studies using standardized diagnostic criteria, objective Eustachian tube assessment, and serial weight measurements are required to determine whether the rate of weight loss predicts PET development.

## Introduction and background

The Eustachian tube serves as the crucial link between the middle ear and the nasopharyngeal space. In its normal state, the Eustachian tube remains closed, only opening briefly during actions like swallowing or the Valsalva manoeuvre. This closure plays a vital role in protecting the middle ear against potential risks such as nasopharyngeal secretions and the transmission of acoustic energy from the nasopharynx, which can be generated during activities like self-vocalisation and breathing [1]. To further understand the pathophysiological consequences of a patulous Eustachian tube (PET), it is imperative to understand the anatomy and physiological function of the Eustachian tube. 

Anatomy and physiology

**Figure 1:**
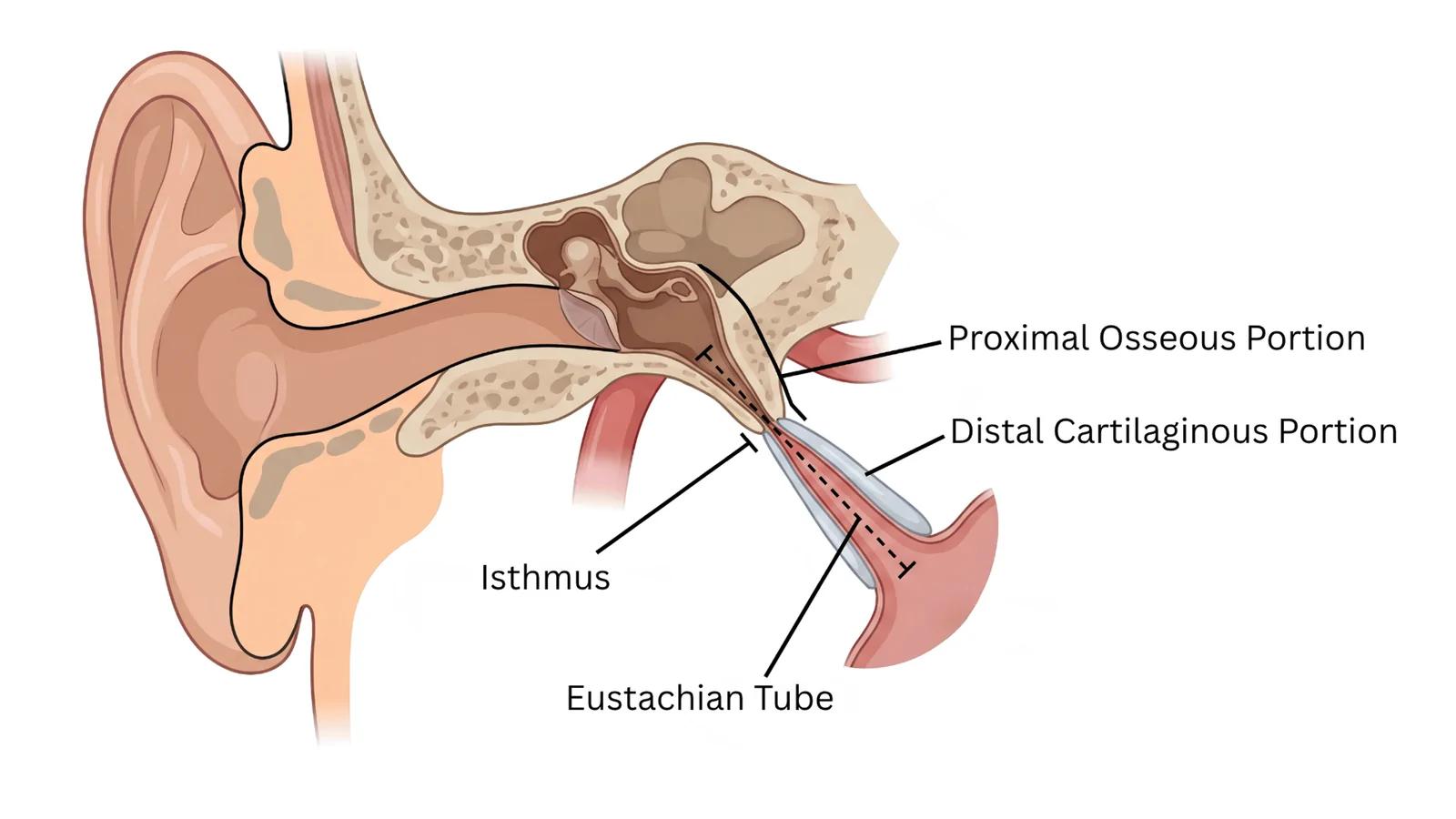
Gross anatomy of Eustachian tube demonstrating 1:2 ratio of proximal osseous portion and distal cartilaginous portion Image credit: Created in BioRender. Almosawi, S. (2026) https://BioRender.com/kdzaryk

The Eustachian tube is an essential structure that connects the middle ear cleft to the nasopharynx, with an average length of 31-38 mm [2]. Anatomically, it is composed of two segments in an approximate 1:2 ratio: a proximal osseous portion, which is embedded within the petrous temporal bone, measuring around 12 mm, and a distal cartilaginous portion, with a length of 24 mm, that opens into the nasopharynx as the torus tubarius, as seen in Figure 1 [1-3].

The isthmus, which is the narrowest point of the tube formed by the junction of the two sections, acts as a physiological barrier by separating the external environment from the middle ear [1]. From this point, the tube proceeds obliquely forward, downward, and medially, forming an angle of roughly 30-45° with the sagittal and transverse planes [2]. 

**Figure 2:**
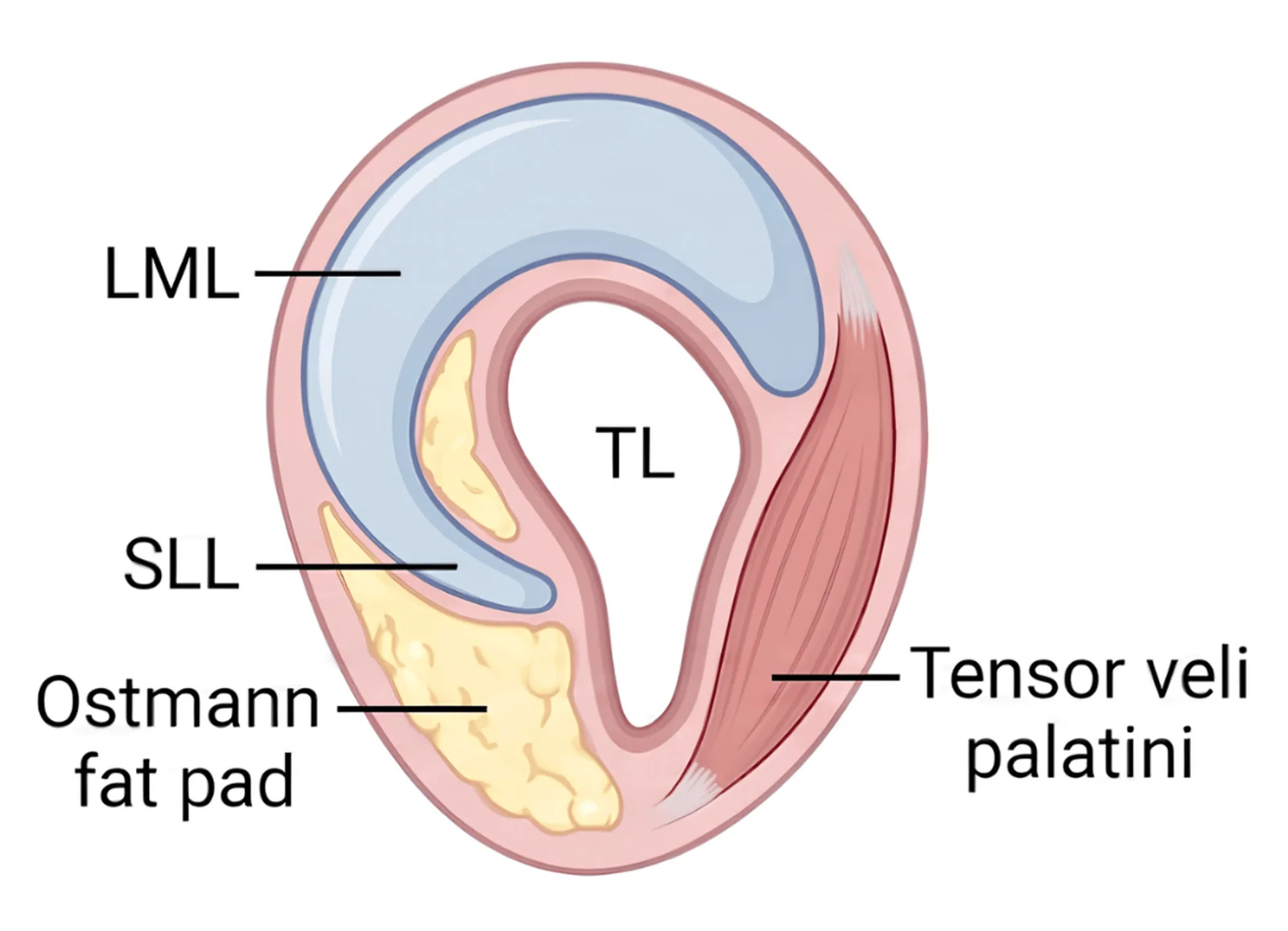
Cross-section of the cartilaginous portion of the Eustachian tube LML: larger medial lamina; SLL: smaller lateral lamina; TL: tubal lumen [6]. Image credit: Created in BioRender. Almosawi, S. (2026) https://BioRender.com/nxhy66s

In terms of anatomy, the bony part is adjacent to multiple structures with clinical significance including the glenoid fossa of the temporomandibular joint laterally and inferiorly, dura mater of the middle cranial fossa superiorly, and the internal carotid artery medially [2]. In addition, the bony section of the Eustachian tube is separated from the surrounding tissues by the mucous membrane which consists mainly of ciliated pseudostratified columnar epithelium intermixed with mucus producing goblet cells (Thesis: Fallstroem JM. Eustachian Tube Dysfunction: Pathophysiology, Diagnostics and Treatment Modalities. A Literature Review: 2026). The cartilaginous portion of the tube has a slightly different anatomical structure from the bony segment. Its cross-sectional outline can be described as “hook-shaped” consisting of a larger lamina on the medial side and smaller one on the lateral side [2]. The lateral end of the cartilage is surrounded by the Ostmann fat pad and the bulk of the lateroinferior wall of the tube is formed by the tensor veli palatini (TVP) muscle as shown in Figure 2 [2,4,5].

The Eustachian tube is acted on by four key muscles: the TVP, levator veli palatini (LVP), salpingopharyngeus, and tensor tympani. Each muscle contributes differently depending on the state, location, and function of the tube. As seen in Figure 2, the TVP muscle is the primary dilator that moves the lateral cartilaginous lamina posteriorly and laterally with activities like swallowing, yawning, and jaw movement. The LVP muscle is a synergist that elevates the soft palate and rotates the medial cartilage although it cannot dilate the tube alone. The salpingopharyngeus muscle contributes minimally to the dilation process, whereas the tensor tympani muscle, positioned in close proximity to the tube, has a limited direct involvement in its opening mechanism and mainly serves to protect the ear from loud noises (Thesis: Fallstroem JM. Eustachian Tube Dysfunction: Pathophysiology, Diagnostics and Treatment Modalities. A Literature Review: 2026) [1,2,4,5]. 

The functional significance of this anatomical structure is in its participation in three main functions: ventilation, drainage, and protection. While at rest, the cartilaginous part remains closed due to the effects of both cartilaginous elasticity and the resting tone of the Ostmann fat pad. In contrast, the bony segment is always open and serves as a passive air channel. Intermittent contraction of the peritubal muscles, mainly the TVP, causes the cartilaginous section to open and permits the passage of air to the middle ear, resulting in pressure equilibration on the tympanic membrane and preservation of hearing. The same ciliated epithelium and contraction of muscles involved in ventilation are responsible for drainage of the secretions from the middle ear towards the nasopharynx because of the oblique orientation of the tube downwards. During the time of the closed state of the tube, the sterile middle ear is protected from secretions, bacteria, and sound transfer from the nasopharynx [7,8]. When this protective closure of the Eustachian tube is compromised, patients may develop a condition known as PET [9].

Disease definition and epidemiology

In contrast to its normal state, a PET represents a chronically patent tube and therefore allows the persistent patency, pressure changes, and sound from the nasopharynx into the middle ear (Thesis: Fallstroem JM. Eustachian Tube Dysfunction: Pathophysiology, Diagnostics and Treatment Modalities. A Literature Review: 2026) [10,11]. As such, patients affected by PET present with a myriad of symptoms such as transmission of one’s sound to themselves (autophony), sensations of aural fullness, pulsatile tinnitus, subjective hearing loss (hypoacusis), and otalgia [10-12]. Some patients also experienced clicking noises and problems with equalization of pressure while diving and flying. In most cases, the signs are intermittent and can appear as a result of physical activity, speaking, or use of decongestants, and are relieved by certain head-lowering maneuvers, and nasal congestion or gentle jugular venous compression [10,11]. Furthermore, it is worth noting that many patients initially experience obstructive Eustachian tube dysfunction, which can progress to patulous dysfunction over time. This progression may be attributed to the atrophy of the luminal mucosa and submucosa of the Eustachian tube, a phenomenon that also occurs within the nasal and sinus passages [12]. PET was believed to be uncommon, but studies have found a prevalence of approximately 0.3% to 6.6% in the total population. It exhibits a notable predominance among female subjects [12,13].

Diagnosis and management

The diagnosis of PET remains challenging due to the lack of a universally accepted gold standard diagnostic method as highlighted by multiple studies [12,14]. Currently, a wide range of diagnostic methods are used to diagnose PET ranging from clinical diagnoses to the use of tubomanometry [15]. A systematic review was performed to further standardize diagnostic approaches for PET and concluded that there is a persistent lack of guidelines and a wide spectrum of diagnostic challenges [16]. In 2012, the Japan Otological Society (JOS) proposed diagnostic criteria to diagnose PET, where a "definite PET" diagnosis requires aural symptoms, tubal obstruction procedures, and objective findings, while "possible PET" requires two out of the three [12,16]. However, as of the date of this article, a universally standardized diagnostic criterion is yet to be introduced. 

Similar to the diagnosis of PET, it is as challenging to find universally accepted guidelines for its management as different approaches to treatment were found in published literature. Conservative management including lifestyle modification consisting of rehydration and the avoidance of rapid weight loss was deemed to relieve the symptoms of PET though temporarily [17]. Other non-surgical management options were reported to be effective such as self instillation of physiological saline solution, mucus thickening agents, and topical irritants to induce mucosal edema of the pharyngeal orifice [17]. However, these management options fail to correct the underlying pathophysiology of this disease. Surgical options for definitive management of resistant PET include the Kobayashi plug (used in Japan) which involves the insertion of a 23 mm length plug through the tympanic membrane allowing the partial blockage of the open space without fully closing the Eustachian tube. The Kobayashi plug was found to be 82.1% successful in a trial consisting of 28 patients receiving the plug with 23 experiencing effective outcomes [17]. Autologous fat injection (a less invasive procedure) was found to have the most promising results with a high success rate in a study involving 28 patients where 20 experienced major improvement in their symptoms, six with only slight improvement and two patients remained unchanged [18]. However, there is limited evidence exploring the effectiveness of conservative and surgical options in the management of PET or provide adequate follow-up to evaluate their long-term impact on patient outcomes. As such, the current management remains patient tailored and would require further studies to establish guidelines. 

Aim of the study

Across the literature, weight loss is repeatedly identified as a risk factor for the development of PET, with an increasing association with bariatric surgery patients. Other reported associations include pregnancy, malignancy, and eating disorders/malnutrition. In this comprehensive review, we aim to examine the role of bariatric surgery as a potential risk factor for the development of PET, as well as highlight all other risk factors documented across literature.

Introduction to bariatric surgery

Bariatric surgery is a mode of weight loss that is categorised as a minimally invasive surgery technique that requires a broad range of surgical skills to conduct with minimal exposure to postoperative complications [19,20]. It has been widely accepted as an appropriate surgical intervention in patients with obesity [21]. This approach to treatment also reduces the risk of cancer, osteoarthritis, and urinary incontinence in comparison to non-surgical treatment. These procedures are slowly growing into the field of surgery, and as of 2018, almost 250,000 procedures were conducted annually in the United States, where 61% of primary procedures were sleeve gastrectomies [21].

Indications for Surgery

In accordance with the criteria established by the National Institutes of Health, individuals with a body mass index (BMI) exceeding 40 kg/m² or those with a BMI greater than 35 kg/m² with related comorbidities become eligible for bariatric surgery. Contraindications for the procedure include severe heart failure, end stage lung disease, active cancer and cirrhosis with portal hypertension [21]. Type 2 diabetes, hypertension, and dyslipidaemia are clinical conditions in which bariatric surgery is indicated. Patients with type 2 diabetes who went through bariatric procedures had better outcomes than patients who relied on pharmacological approaches for glycaemic control and remission [22]. Thus, bariatric surgery remains as an excellent surgical treatment option for patients struggling with various chronic diseases. 

Bariatric Surgery Techniques

**Figure 3:**
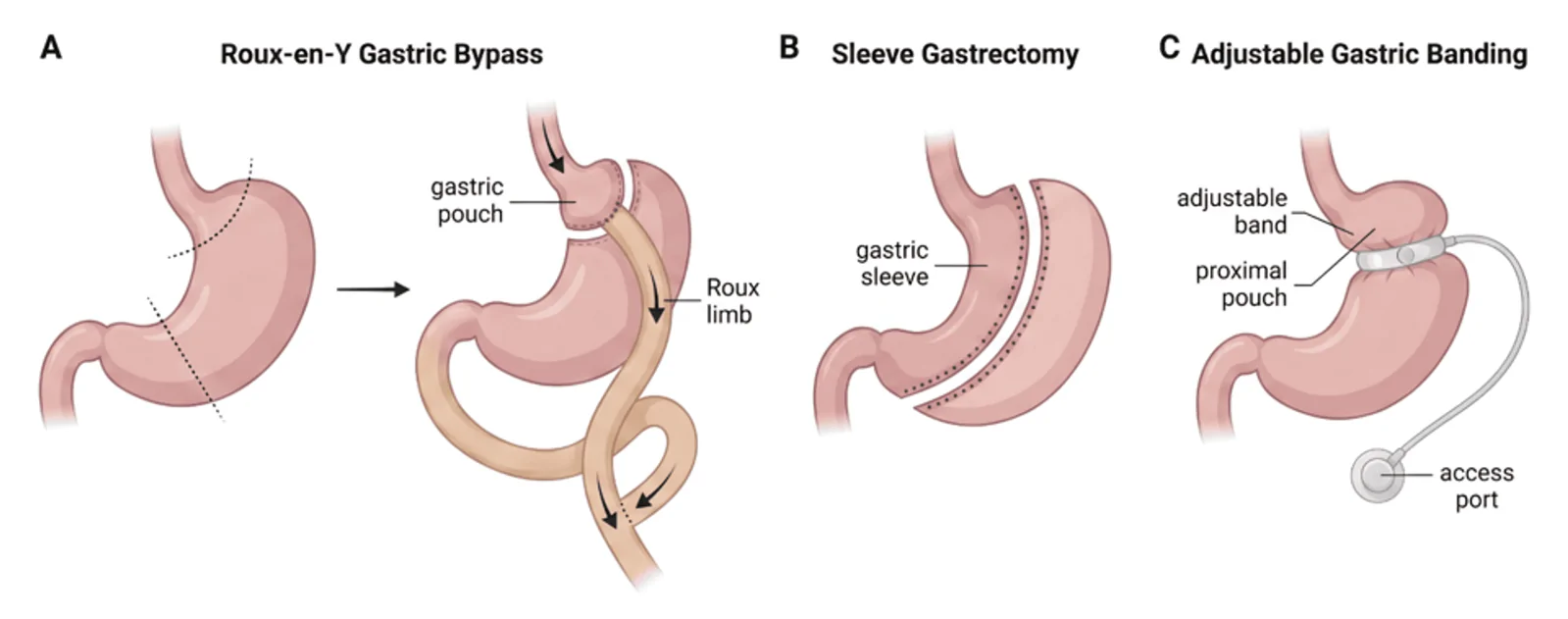
Three common techniques used for bariatric surgery A: Roux-en-Y Gastric Bypass, B: sleeve gastrectomy, C: adjustable gastric banding. Image credit: Created in BioRender. Almosawi, S. (2026) https://BioRender.com/gnn4j8s

Three major bariatric surgery techniques are currently used for weight loss with the most common being sleeve gastrectomy. In this procedure, a narrow tube-like structure is the end result after the removal of the gastric fundus and the greater curve of the stomach. The Roux-en-Y gastric bypass has been increasing in popularity in recent years. It involves creating a pouch from the stomach and connecting it to the jejunum bypassing part of the stomach and the small intestine in the process. Adjustable gastric banding is also a method used where a band is placed laparoscopically and can be adjusted via an access port [19]. Figure 3 illustrates the most common bariatric surgery techniques.

Postoperative Complications

Postoperative complications of bariatric surgery are considered well studied especially over the recent years since the introduction of the laparoscopic approach. Major postoperative complications of bariatric procedure include cardiac, pulmonary, nutritional, and peritoneal complications. Bleeding, infection, thrombosis and failure of operation are generally considered across all types of bariatric surgery. Stenosis of the newly formed tube is a known postoperative complication, where an endoscopic intervention with balloon dilatation of the stenosed region is often sufficient for correction. Additionally, some published reports discuss the prescence of auditory symptoms post bariatric surgery. However, the development of autophony post bariatric surgery is poorly recognized and understated [23]. 

## Review

Methodology

Several databases were used to ensure a high-quality literature search. Due to limited evidence, articles were included from inception until August 23, 2026, using PubMed Medline, Google Scholar, and Scopus, using these keywords: Patulous Eustachian Tube (PET), Bariatric Surgery, weight loss, tubal dysfunction. Only published complete articles were included. The total number of articles included in this study is 38.

PET following bariatric surgery

In an analysis of the literature, it was found that limited research exists concerning the relationship between bariatric surgery and PET. However, isolated observational and case reports suggest that PET symptoms may worsen following bariatric surgery. These reports detail instances where patients (predominantly female patients) undergoing weight loss surgery encountered an escalation in symptoms related to PET. One of the very first researchers to observe this phenomenon was Letti et al. [24]. The study demonstrated the presence of tubal dysfunction in eight patients who lost an average of 15 kg over 45-65 days. In a study carried out by Muñoz et al., the findings demonstrated a noteworthy occurrence of PET among individuals who have undergone bariatric surgery, and this occurrence appears to be connected to the speed and extent of weight loss. A total of 141 patients underwent bariatric surgery with 21.28% of patients developing PET [25]. This finding is common across literature, as Kinasz et al. observed that roughly 18.75% of the patients who underwent bariatric surgery in the sample had symptoms compatible with PETs [26]. When compared to the asymptomatic group, the symptomatic individuals had lower a preoperative weight and BMI, which suggests an association between the occurrence of symptoms and decreased body weight and BMI before surgery. However, no further studies indicate any association between preoperative weight and the presence of symptoms, making this finding an abnormality. Another observational study conducted in Saudi Arabia demonstrated that 20.22% of patients post-bariatric surgery displayed clinical symptoms of PET [13]. All who presented with symptoms were female patients. Furthermore, Pascoto et al. found 14 of the 19 patients who denied any auditory symptoms pre-operatively were found to have auditory symptoms up to six months postoperatively [27]. Overall, research seems to suggest that patients with a history of rapid weight loss are more likely to develop PET symptoms.

Physiological Mechanism

The mechanism of how bariatric surgery can contribute to the development of PET is inconclusive and hypothetical. However, the collective conclusion suggests that the rapid loss of fat around the Eustachian tube causes it to remain patent in many patients. The loss of peritubal Ostmann fat pad results in reduced peritubal structural support limiting the passive closure of the cartilaginous Eustachian tube. This persistent luminal patency results in symptoms such as autophony and aural symptoms. A study by Pascoto et al. hypothesized that rapid weight loss due to the surgery causes the loss of the peritubal pad directly affecting the integrity of the Eustachian tube [27]. Another study by Kinasz et al. concluded that the loss of the Ostmann fat pad is the most likely driver of its patency after rapid weight loss. However, there was no objective measure pertaining to the fat pad. As such, it was hypothesized that the rapid loss of weight contributed to the loss of competency of the Eustachian tube [26]. Additionally, the apparent female predominance in PET following bariatric surgery also remains unexplained across all literature. Although speculative, one possible explanation might be the influence of hormonal factors on peritubal adipose tissue or on Eustachian tube function. Further studies are required to determine whether hormonal differences contribute to the observed female predominance and to clarify the mechanisms underlying this association.

Bariatric surgery or Rapid Weight Loss?

The claim that rapid weight loss contributes to the development of PET is also demonstrated in patients using medical treatments to rapidly decrease their weight. Two studies were published regarding patients using GLP-1 agonists for weight loss purposes. One study consisted of seven patients who developed symptoms of PET within four to 10 months of GLP-1 agonist use with weight loss ranging between 8.2% to 18.7% from baseline [28]. A study by Pak et al. mentions that two cases of PET-associated symptoms following GLP-1 receptor agonist use were reported, with nasal endoscopy demonstrating loss of tissue bulk of the anterior and posterior Eustachian tube cushions [29]. These findings suggest that PET may be associated with rapid weight loss rather than being exclusive to bariatric surgery. However, the limited number of reported cases and lack of comparative studies render it difficult to have definitive conclusions regarding this association.

Additionally, an important unexplored area related to PET is the rate or magnitude of weight loss and associations with disease progression. Although evidence highlights rapid weight loss postoperatively or pharmacologically as being associated with PET development, the available studies do not consistently quantify weight loss in a manner that allows the rate/magnitude to be examined. A rate-dependent relationship between weight loss and development of PET has not yet been established. 

Limitations of Current Evidence

The current evidence is limited by the predominance of small observational studies and case reports, a variety of different diagnostic criteria, and the absence of prospective control groups. In addition, many studies rely on subjective symptoms rather than objective measures of Eustachian tube patency or peritubal adipose tissue. Therefore, the reported association should not be interpreted as evidence of causality. Further research is required to establish whether rapid weight loss is directly responsible for PET development. Additionally, it is crucial to examine the rate of weight loss at which patients develop Eustachian tube patency. Prospective studies should incorporate standardized diagnostic criteria and objective assessments of Eustachian tube function as well as serial measurements of body weight. 

Furthermore, the proposed role of Ostmann’s fat pad remains theoretical, as no studies demonstrated objective quantifiable changes in peritubal adipose tissue before and after weight loss. Imaging or endoscopic studies, although costly, can assess changes in peritubal tissue volume alongside objective measures of Eustachian tube function. This would help determine whether this proposed mechanism is responsible for PET following rapid weight loss.

Future prospective studies should evaluate patients before and after bariatric surgery using standardized objective diagnostic criteria for PET, serial measurements of weight and BMI, and an objective assessment of Eustachian tube function. Correlating all these factors will help in establishing whether a dose-response relationship exists. Studies should also investigate potential hormonal factors that may contribute to the apparent female predominance.

Other risk factors and disease associations

Although the association between PET and bariatric surgery remains inconclusive and not well studied, several other conditions have been associated with the development of PET. Reviewing these established risk factors provides important context for understanding the potential pathophysiological mechanism underlying PET development and helps determine whether the association with bariatric surgery is specific to the surgery itself or related to shared factors such as weight loss and alterations in peritubal tissue. Additionally, focusing on these aspects might lead to establishing definitive diagnostic and management guidelines. The following section summarizes the principal risk factors and associated conditions reported in the literature.

Weight Loss in Other Conditions

As previously demonstrated, rapid weight loss has been identified as a major contributor to the development of PET. This correlation is not specific to medical/surgical interventions in voluntary weight loss, as one study explored the prevalence of PET in anorexia nervosa patients. PET was strongly suspected in 16 out of 73 patients in the study [30]. With anorexia nervosa, severe and prolonged caloric restriction leads to significant loss of body fat, including the fat surrounding the Eustachian tube, and PET has been reported as a recognized complication of this condition. Another study by Hollis et al. found that autophony was commonly reported in patients with severe eating disorders, as 43 out of 101 patients developed these symptoms. Additionally, lower serum prealbumin levels and lower body weight as measured by percentage of ideal body weight were also associated with the presence of autophony [31]. However, the study had several limitations, including the absence of a validated PET-specific symptom assessment tool and incomplete usage of an objective Eustachian tube functional testing. In that study, the primary method for data collection was a self-reporting survey in which patients reported autophony. Furthermore, the lack of a control group limited comparison of PET prevalence, while the absence of follow-up data prevented assessment of whether symptoms improved with weight restoration. Overall, evidence supporting this association remains limited, with relatively few studies specifically investigating PET in patients with eating disorders. The available evidence is predominantly observational, limiting the strength of the association and preventing definitive conclusions regarding causality. Nevertheless, the occurrence of PET in the context of non-surgical and non-pharmacological weight loss provides further support for the hypothesis that the loss of peritubal adipose tissue may contribute to the development of PET. 

Pregnancy

Pregnancy has been proposed across literature as a risk factor for PET, with hormonal and metabolic changes potentially contributing to Eustachian tube patency independently of rapid weight loss. Several mechanisms have been proposed to explain this association across literature. One hypothesis suggests that increased fat metabolism during pregnancy may result in depletion of fatty tissue surrounding the Eustachian tube, which potentially reduces the structural support required to maintain tubal closure leading to patency of Eustachian tube. This mechanism is similar to the ones described pertaining to rapid weight loss and might suggest that alterations in peritubal adipose tissue represent a common pathway across different physiological states. Another hypothesis proposes that increased oestrogen levels during pregnancy might alter Eustachian tube mucosal lining resulting in thinning of mucosal lining and reduced resistance to tubal relaxation [32]. A further hypothesis also incorporates the established effects of estrogen on connective tissue during pregnancy, as an experimental study demonstrated that oestrogen contributes to softening of pelvic cartilage and increased elasticity, raising the possibility that similar hormonal effects on cartilage of the Eustachian tube could increase its compliance and predispose it to remaining patulous [33]. These proposed mechanisms are not necessarily mutually exclusive and may act synergistically. Overall, definitive conclusions are difficult to draw given the limited understanding of the underlying physiological mechanisms [32]. Additionally, the evidence supporting pregnancy as a risk factor for PET remains limited and is largely derived from older studies. No modern studies have validated these proposed mechanisms. The relative contribution of hormonal, metabolic, and anatomical changes therefore remains uncertain. Further prospective studies are required to clarify the relationship between pregnancy-related physiological changes and Eustachian tube patency to determine the extent to which hormonal factors contribute to the development of PET.

Radiotherapy and Malignancy

Radiotherapy represents another potential risk factor for PET particularly in patients treated for nasopharyngeal carcinoma and other head and neck malignancy [34-36]. Evidence from long-term follow-up studies suggests that PET may develop several years after irradiation, indicating that the effects of treatment on Eustachian tube function may evolve over time. A longitudinal study by Young et al. demonstrated that 12 of the 20 ears (10 patients total) demonstrated a PET 10 years after irradiation. The researchers also found that the occurrence of a patulous tube was independent of radiation dosage and related to interval since irradiation, suggesting that atrophy of the peritubal tissues may contribute to the development of PET [34]. Similarly, another long term study identified that half of the ears examined 10 years after irradiation developed PET. These findings suggest that radiation-associated changes in Eustachian tube function may be progressive and may become apparent only several years following treatment with no dose-dependent relationship being demonstrated. 

A further study by Ohkoshi et al. demonstrated PET conditions in patients undergoing concurrent chemoradiotherapy for head and neck squamous cell carcinoma. PET conditions were defined as per the diagnostic criteria of Japan Otological Society. In that study, PET was identified in 22.2% of ears before treatment and 27.8% of ears three months after concurrent radiochemotherapy. However, pre-existing PET was the only factor significantly associated with PET following treatment. Therefore, these findings do not establish chemoradiotherapy as an independent cause of PET. Furthermore, the relatively short three-month follow-up period may have limited the ability of the study to detect delayed structural or functional changes, particularly given the evidence from long term studies demonstrating that PET may become apparent years after irradiation [35].

The mechanisms underlying radiation-associated PET remain incompletely understood. Proposed mechanisms included atrophy or fibrosis of tissues surrounding the Eustachian tube and loss of peritubal supporting tissue. However, these changes may occur alongside substantial weight loss and cachexia commonly associated with head and neck malignancy, making it difficult to distinguish the effects of radiotherapy from those of systemic weight loss. This is particularly relevant given the established association between rapid weight loss and PET discussed earlier in the review [35]. 

Histopathological findings provide further insight into the potential mechanisms underlying radiation-associated PET [37]. An isolated case report was published on a temporal bone specimen from a patient with nasopharyngeal carcinoma who had undergone radiotherapy and experienced substantial weight loss. A PET was observed and confirmed via histopathology as there was fibrous tissue replacement of the surrounding structures including Ostmann fat pad, LVP muscle, and submucosal glands around the Eustachian tube cartilage. The authors proposed that the tumour invasion, radiation-induced fibrosis, and atrophy of adjacent submucosal and lymphoid tissue may have contributed to widening of the Eustachian tube's lumen. In addition, the substantial weight loss and cachexia experienced by the patient were considered potential contributors to the loss of peritubal supporting tissue. These findings suggest that radiation-associated PET may arise through the interaction of local treatment-related tissue changes and systemic rapid weight loss rather than being attributable to radiation exposure alone [37].

Nevertheless, the evidence remains limited, as histopathological findings are derived from an individual case and therefore cannot establish a causal relationship between radiotherapy and PET. Furthermore, the concurrent presence of tumour involvement and substantial weight loss makes it difficult to determine the relative contribution of each factor to the observed anatomical changes. Further longitudinal studies are required to clarify the mechanisms underlying radiation associated with PET and determine whether radiotherapy represents an independent risk factor or acts synergistically in tandem with rapid weight loss.

Clinical Implications

PET should be considered in patients who develop new otological symptoms following rapid weight loss. These symptoms may emerge following bariatric surgery and may also occur in other settings associated with rapid weight loss supporting the possibility that the rate of weight loss might be the driving factor rather than its mechanism. 

Preoperative counselling and postoperative follow-up could include awareness of PET as a potential otological complication of substantial weight loss. Patients should be advised to report new-onset autophony, persistent aural fullness, or other unusual auditory symptoms after surgery. This may facilitate earlier recognition and referral to ear, nose and throat (ENT) services, particularly in female patients with rapid postoperative weight loss. 

Importantly, objective confirmation should be sought as PET can be intermittent and symptoms may not always correlate with examination findings. Assessments may include otoscopy with observation for respiration-related tympanic membrane movement, positional assessment, nasopharyngoscopy, dynamic tympanometry, or sonotubometry. However, the absence of visible tympanic membrane movement should not exclude PET. Further structural studies can be incorporated in patients with high suspicion of PET such as magnetic resonance imaging (MRI) to examine structural integrity of the Eustachian tube.

Overall, the clinical significance of this association lies less in establishing PET as a common complication of bariatric surgery and more in improving recognition of an underdiagnosed condition in patients undergoing substantial weight loss.

## Conclusions

In conclusion, the available evidence suggests an association between rapid weight loss and the development of PET following bariatric surgery. It is insufficient to establish bariatric surgery itself as an independent risk factor. However, the occurrence of PET following other forms of rapid weight loss, including pharmacological agents and severe malnutrition, suggests that the magnitude and rate of weight loss may be more important determinants than the method by which it is achieved.

In our review, we explored potential pathophysiological mechanisms for PET, in particular the loss of peritubal adipose tissue (Ostmann’s fat pad), and highlighted it as the most plausible proposed mechanism through which rapid weight loss may impair Eustachian tube closure. Nevertheless, this mechanism remains hypothetical due to a substantial lack of longitudinal evidence. Moreover, the apparent female predominance and the potential contribution of hormonal factors remain inadequately understood. Future research should target this research gap, with more evidence needed to understand what factors drive the pathophysiology of Eustachian tube impairment.

Overall, PET should be recognized as a potential but under-recognized consequence of substantial weight loss. Clinicians should maintain a high index of suspicion when patients develop autophony, aural fullness, or other characteristic symptoms following bariatric surgery or other states of rapid weight loss. Future prospective studies using standardized diagnostic criteria, objective assessment of Eustachian tube function, serial measurements of weight loss, and assessment of peritubal tissue are required to determine whether a dose-response relationship exists and to clarify the mechanism underlying this association.
